# Spread and impact of fall armyworm (*Spodoptera frugiperda* J.E. Smith) in maize production areas of Kenya

**DOI:** 10.1016/j.agee.2019.106804

**Published:** 2020-04-15

**Authors:** Hugo De Groote, Simon C. Kimenju, Bernard Munyua, Sebastian Palmas, Menale Kassie, Anani Bruce

**Affiliations:** a International Maize and Wheat Improvement Centre (CIMMYT), Nairobi, Kenya; b Agri-food Economics Africa, Nairobi, Kenya; c Icipe, Nairobi, Kenya

**Keywords:** Maize, Fall armyworm, Loss, Community survey, Focus group discussions

## Abstract

Fall armyworm (FAW), one of the most important pests of maize in Latin America, suddenly appeared in Africa in 2016 and spread rapidly. Estimates of crop losses due to FAW are essential in order to compare the impact of these losses with the cost of controlling FAW and advise appropriate technology dissemination and policy. In this study, therefore, crop losses due to FAW in 2017 and 2018 were estimated in all the maize production areas of Kenya. Data were collected during June and July 2018 through 121 group discussions with 1439 farmers, separately with men (697) and women (742), in communities that were randomly selected to represent the major maize growing areas. The results showed that most participants (82%) could correctly identify the FAW from pictures. By 2016, FAW was observed by more than half of the communities (53%), with most of the other half first observing FAW in 2017. The proportion of farmers affected by FAW substantially increased, from the long rains of 2017 (63%) to the long rains of 2018 (83%), and in all zones except for the high tropics and moist mid-altitudes. However, the percentage of loss experienced by affected farmers decreased slightly, from 54% in 2017 to 42% in 2018. In 2017, the low- and medium-potential maize-production areas were the most affected, with losses of >50%, with high-potential areas facing losses of about 30%, resulting in a total loss of 37% for the whole country. In the main 2018 season, losses in the low- and medium-potential areas were less – about 20%, but the high-potential areas were now more affected, leading to a total estimate of 33%. We conclude that FAW has suddenly become a major pest in Kenya, causing losses of about a third of the annual maize production, estimated at about 1 million tonnes.

## Highlights

• Discussions on FAW were conducted in 121 communities with 1439 farmers in Kenya.
• Most participants (82%) could correctly identify FAW from pictures.
• In 2016 FAW had been observed by 55% of the communities, in 2017 by 98%.
• By 2018, FAW affected 83% of maize farmers, causing losses of 33%, 1 million tonnes.

## Graphical abstract

**Figure:**
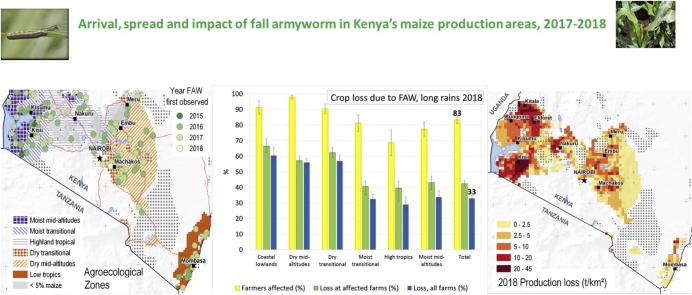

## 1 Introduction

Fall armyworm (FAW), *Spodoptera frugiperda* (J.E. Smith) is a lepidopteran polyphagous pest native to tropical and subtropical America, where it is one of the most important maize pests, for example in Central America and Brazil (Cruz et al., 1999; Sarmento et al., 2002). It belongs to the genus *Spodoptera*, known as armyworms, the group of Noctuidae that causes the highest monetary losses to agriculture worldwide (Pogue, 2002). The pest appeared suddenly in Africa in early 2016, when it was first reported in central and western Africa (Goergen et al., 2016) from where it spread very quickly. By 2017, it was found in most of sub-Saharan Africa (Day et al., 2017), threatening food security (Devi, 2018). Some of the factors that helped FAW to spread quickly over the continent were its propensity to attack a wide range of crops (Huesing et al., 2018); its ability to produce many eggs (Sparks, 1979); its preference for maize, the major cereal crop in Africa (Devi, 2018), and its ability to migrate over long distances (Rose et al., 2012). Since the arrival of the FAW in Africa, several reports, overviews and guidelines have been published but, so far, there has been no systematic, quantitative nation-wide study in any of the affected countries.

As FAW was a new pest to Africa, the first set of publications on its invasion were guides, documenting the extent of the problem and its potential impact, while at the same time proposing management measures. These publications came from international organizations working in the field, including the Food and Agriculture Organization (FAO, 2017, 2018) and the International Maize and Wheat Improvement Centre (Prasanna et al., 2018). These guides were based mainly on expert opinion and literature review.

At the same time, several studies tried to estimate the impact of FAW, in particular the crop losses that it causes. The first study, based on surveys, estimated that FAW had the potential to cause maize yield losses from 8.3 to 20.6 million tonnes per annum (21–53% of production), if left uncontrolled (Abrahams et al., 2017; Day et al., 2017). A second study, based on farmers’ estimates, estimated FAW infestation rates of 32% in Ethiopia (with yield reductions of 934 kg/ha) and 47% (1381 kg/ha) in Kenya (Kumela et al., 2018). The only direct measurement of FAW loss in Africa, from Zimbabwe, estimated the losses in 2018 at 11.6%, but only from two district (Baudron et al., 2019).

Because of the high potential losses caused by FAW, a range of control methods have been proposed (Hailu et al., 2018; Kumela et al., 2018; Midega et al., 2018; Harrison et al., 2019). To judge and compare these different options, however, it is not enough to study their efficacy but, more importantly, their costs need to be compared to their benefits. After all, farming is an economic activity, even among semi-subsistence farmers in Africa. While calculating pesticide costs is fairly straightforward, estimating the labor costs for their application is more difficult, as is estimating environmental costs (Leach and Mumford, 2008). However, the most challenging aspect of the economic analysis of pest control is to estimate the crop loss due to the pest and the reduction in the loss due to the control.

Crop loss estimates have a long history, and world-wide estimates have been produced at regular intervals (Ordish, 1952; Cramer, 1967; Oerke et al., 1994; Oerke, 2006). The methodology of crop-loss assessment was developed and fine-tuned through a series of workshops and conferences (Chiarappa, 1971; Teng, 1991). The FAO definition of crop loss is the reduction in yield attributed to the pest, or the difference between attainable yields and actual yields, usually expressed as a percentage (Walker, 1983). Yield loss can be estimated in different ways: directly, through experiments or field surveys (Walker, 1983); indirectly, by establishing the link between pest incidence and yield loss (Walker, 1991); through expert opinion (Teng, 1990); through farmer estimates (De Groote, 2002); or through community surveys (De Groote et al., 2016). Despite these available methods, crop losses due to pests remain notoriously difficult to estimate. Firstly, pest incidences and the resulting losses can be highly variable, both over space and over time. Secondly, it is often difficult to establish good control plots, where only the pest understudy is eliminated and not other pests, that can then be compared to the infested plot. Consequently, direct and systematic loss assessments are rare.

In this study, we used community surveys to estimate crop losses caused by FAW in the major maize-growing areas of Kenya. This method was chosen for practical reasons; shortly after the start of the FAW invasion in Kenya, we were able to add questions on FAW, at little extra cost, to an already-planned survey on maize production constraints in randomly selected communities in the major maize production areas of Kenya. By engaging men and women in group discussions, we were able to obtain information about their knowledge of FAW, and their observations on the arrival of the pest in their communities. Further, we asked participants to estimate the proportion of farmers affected by FAW, and the loss caused in the maize fields of those affected farmers. To our knowledge, this is the first systematic and country-wide assessment of the impact of FAW in Africa.

## 2 Methods

### 2.1 Yield loss estimation

Crop yield loss is defined as the difference between attainable yield *Y_a_* and actual yield *Y,* and expressed as a proportion of the attainable yield percentage $r=\;\frac{\left(Ya\;-\;Y\right)}{Ya}\times \;100$. Instead of trying to measure *Y_a_* and *Y* directly, we asked farmers, during group discussions, to estimate the proportion of farmers affected (*F_a_*) in their community, and the loss (in %) experienced by the affected farmers (*L)*. Total loss in the community was then calculated as *r = F_a_ x L.* As the communities were selected randomly from the major maize production zones, average yield losses could be multiplied by the estimated maize production in each zone to estimate maize quantities lost. The method was previously used to estimate crop yield loss and its distribution caused by the maize lethal necrosis (MLN) disease (De Groote et al., 2016), and was based on previous experience to assess the importance of different maize pests through group discussions and geographic information systems (De Groote et al., 2004b).

### 2.2 Design of the community survey

A community survey was designed to mirror the maize lethal necrosis (MLN) survey of 2013 (De Groote et al., 2016). Thus, the same 121 communities that were interviewed in 2013 were targeted. These communities were randomly selected to represent the six main maize production areas in Kenya. The main purpose of the community survey was to assess farmer prioritization of various stresses and to measure the impact of these for the Stress Tolerant Maize for Africa (STMA) project. Prioritization is especially important due to the arrival of new pest problems, in particular the larger grain borer (LGB), MLN disease and the current fall armyworm (FAW). Data were collected through focus group discussions (FGDs). CIMMYT contracted Agri-Food Economics Africa, a research company based in Kenya, to undertake the study.

### 2.3 Development of tools

The development of the questionnaire was a consultative process undertaken during the first half of 2018, involving CIMMYT and partners who had a special interest in FAW. These partners were the International Centre of Insect Physiology and Ecology (*icipe*), the Food and Agriculture Organization (FAO) and the CAB International (CABI), as well as CIMMYT economists and entomologists. Comments from these partners were taken into consideration, and efforts were made to harmonize sections of the tools with those of the partners, such as the FAO’s FAW modules.

The primary goal of the study was to assess the importance of different maize production and storage stresses, as perceived by farmers in the different agroecological zones where maize is produced. A draft questionnaire was developed and tested; this contained general questions on which crops and which maize varieties were commonly grown, with modules to score the importance of biotic and abiotic stresses, coping strategies and access to maize seed. Separate modules discussed various stresses, including FAW, MLN, maize stem borer, Striga weed, maize weevil, larger grain borer, drought, and soil fertility. Due to the realization that responses may vary by gender, some sections of the questionnaire were designed to collect gender-disaggregated information.

The questionnaire was pre-tested for two days, 7^th^ and 8^th^ June 2018, with two communities that were not participating in the survey, one in Machakos County and the other in Embu County. In addition to economists from CIMMYT and Agri-Food Economics Africa, a CIMMYT entomologist and an economist from ICIPE participated in the pre-test. Following the pre-testing, adjustments were made to the questionnaire, and a version developed that was used for training enumerators. This version also formed the basis of the electronic questionnaire designed using the SurveyCTO platform, as enumerator training was based on both paper and electronic questionnaires. After two days of training, the team of enumerators, field supervisors and researchers piloted the survey in Murang’a County. This was followed by a recap to raise and discuss all the issues observed. The team of researchers discussed all additional issues observed during training and piloting, and developed a final version of the questionnaire that was to be used for data collection (Appendix 1). The electronic questionnaire was also updated to reflect the final paper version.

Since the community survey dealt with biotic and abiotic stresses, it was important to have pictures that represented the various biotic stresses (insect pests and diseases) so that the farmers could recognize the specific pest that they were being asked about. In addition, the photos were important in helping to gauge farmers’ awareness of the fall armyworm. CIMMYT entomologists assisted in gathering these pictures and in refining the descriptions of the various stresses. The final version of the pictures was printed and laminated for use in data collection.

### 2.4 Site selection

The survey targeted the same communities that were interviewed for a study in 2013 (De Groote et al., 2016). Each field team was given a list of the communities that they were to interview, with the previously allocated identification number, location details (division, location and sublocation), and contacts of the members who participated in the 2013 FGDs. The contacts in the communities, usually a leader from a farmer group or from the local administration, were each asked to invite between 10–15 maize farmers. They were asked to make sure that both men and women participated, and older as well as younger farmers.

### 2.5 Data collection

Data collection was initially planned to start on February 2018 but was eventually delayed till June 2018. Firstly, some of the study partners wanted to conduct the survey later in the maize season, to better estimate the impact of the stresses, especially from FAW, in the field. The team, therefore, agreed to delay the start of the survey until April 2018. However, it was raining heavily at this time in many parts of the country, so it was resolved to delay the start of the survey until June when the rains have usually subsided.

Ethical clearance for the survey was sought by CIMMYT from CIMMYT’s Institutional Research Ethics Committee (IREC), and the research was cleared for implementation on 11^th^ June 2018 (clearance number IREC 2018.004).

Data collection was undertaken by Agri-Food Economics Africa, which recruited two teams, each consisting of an experienced supervisor and two experienced enumerators. The minimum qualification for supervisors was at least three years of experience in managing or conducting household surveys, as well as having served as enumerators themselves. The minimum qualification for an enumerator was a university degree in agricultural or related sciences. All team members were properly trained in the different aspects of the survey and the questionnaire and participated in the survey pilot as part of the training and preparation.

Data collection took place from 18^th^ June to 28^th^ July 2018 (41 days). Each of the two teams was provided with a car and driver. One of the teams started data collection in western Kenya, covering the high-potential zones, while the other started in the east, covering the coast and the dryland zones. During the fieldwork, Agri-Food Economics Africa researchers visited the teams to check on their operation and strengthen any areas identified as weak.

Each team undertook two FGDs per day on most days as had been planned. Sometimes, however, this was not possible because of various reasons, mainly long distances and heavy rain in some parts of the country. At the end of the data collection, all targeted 121 communities were interviewed, representing 100% coverage with no replacements. In total, 1439 farmers participated, of which 742 women.

### 2.6 Analysis

Kenya does not produce regional maize statistics. To estimate maize production by agroecological zone, we used the definition of the zones as developed by Hassan (Hassan et al., 1998). We compared Hassan’s area- and production statistics with the data from the 2005 and 2010 Spatial Production Allocation Map (SPAM) (Yu et al., 2017; You et al., 2014), and calculated the maize area and production for 2005 and 2010 for the different agroecological zones (AEZs). Next, we looked at the maize production statistics of FAO (FAOSTAT, 2019), and found that during the years before the arrival of the FAW, from 2011 to 2016, these statistics were relatively stable, so we used them to estimate the total maize area (2.16 million ha) and production (3.57 million tonnes) before the FAW. We distributed these totals proportionate to our zonal area and production estimates based on SPAM 2010 (Appendix 2). To estimate the population in each agricultural zone, we used the 2015 population density dataset from WorldPop (www.worldpop.org) (Stevens et al., 2015). Finally, we allocated the annual production data for each zone to the two seasons, proportionate to the distribution found in the household survey undertaken by CIMMYT in 2013.

For the geographic information system (GIS) analysis, a surface of 1 km cells was generated using the estimated percentage of production lost in each community and interpolating using inverse distance weighting (IDW) with a power of 2 and a variable search radius. As an estimate of total maize production in different zones, we used HarvestChoice’s 2010 SPAM (You et al., 2014). We resampled the 2010 SPAM production data from cells of 10 km to 1 km. Multiplying the surface of percentage of production lost with the maize production data resulted in the quantities of maize lost, in tonnes per km^2^.

## 3 Results

### 3.1 Maize production in Kenya

**Fig. 1:**
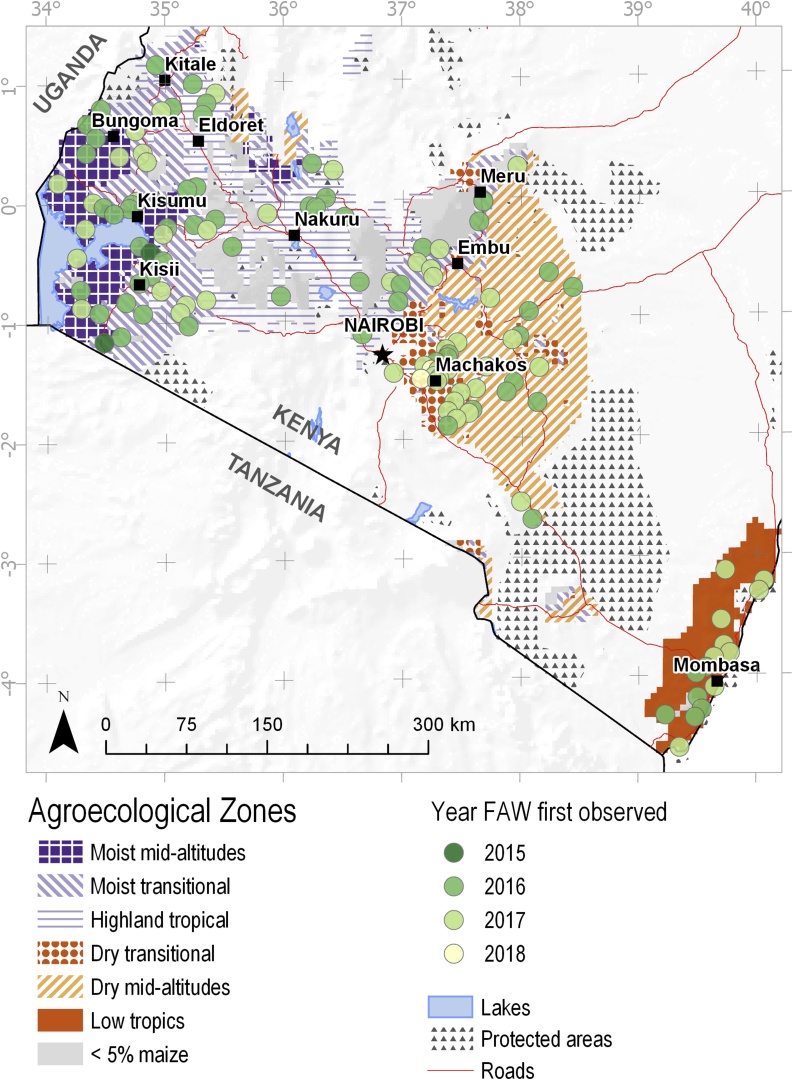
Spread of FAW arrival over time and space.

In Kenya, six maize-growing agroecological zones have been identified (Hassan et al., 1998); these include, from east to west, the lowland tropics (LT) on the coast, followed by the dry mid-altitude- and dry transitional zones around Machakos (Fig. 1, Appendix 2). These three zones are characterized by low yields (around 1 t/ha) and, although they cover 50% of the maize area, they only produce 30% of the maize (Table 1). Further inland, in central and western Kenya, the highland tropics (HT) are found, bordered on the west and east by the moist transitional (MT) zone; both these zones have high yields (more than 2.5 t/ha) and produce about half of the maize in Kenya on 30% of the area. Finally, around Lake Victoria, is the moist mid-altitude (MM) zone, with moderate yields (1.5 t/ha).

**Table 1:** Household maize area and production by season and production zone (from 2013 CIMMYT household survey, numbers in brackets are standard deviations).

	Main season			N	Short season			Annual			Total 2010-2016	
	Production (kg)	Area (ha)	Yield (kg/ha)		Area (ha)	Yield (kg/ha)	N	Prod. (Kg)	Area (ha)	Yield (kg/ha)	Prod. (1000 tonnes)	Prod.(% main season)
Coastal Lowland	507	1.04	574	89	0.97	464	74	817	1.84	544	42	62
	(563)	(0.74)	(566)		(0.69)	(595)		(830)	(1.36)	(493)		
Dry mid altitude	444	1.42	427	180	1.53	592	162	1071	2.80	509	42	41
	(664)	(1.44)	(810)		(1.91)	(950)		(1817)	(2.91)	(800)		
Dry Transitional	648	0.91	1003	126	0.93	867	118	1272	1.78	859	217	51
	(1105)	(1.11)	(2412)		(0.99)	(1015)		(2034)	(2.04)	(1063)		
Moist Transitional	1218	0.72	1476	357	0.57	1176	211	1650	1.06	1409	1465	74
	(3609)	(1.20)	(1670)		(0.90)	(1514)		(4914)	(1.68)	(1575)		
High Tropics	3845	0.98	2439	238	0.31	1308	34	3885	1.02	2454	908	99
	(18,311)	(1.79)	(2409)		(0.29)	(1362)		(18,305)	(1.79)	(2419)		
Moist mid altitudes	713	0.74	1199	238	0.65	985	196	1173	1.27	1099	458	61
	(945)	(0.64)	(1236)		(0.67)	(980)		(1633)	(1.12)	(1087)		
Total maize AEZ	1406	0.92	1494	1228	0.86	903	795	1807	1.48	1301	3131	78
	(8393)	(1.28)	(1859)		(1.18)	(1163)		(8620)	(1.96)	(1651)		
Other areas											428	78
Grand total											3559	78

As Kenya is located on the equator, it has two maize-growing seasons. However, their relative importance differs between zones (Table 1). In the highlands, almost all the maize (99%) is produced in the main season (March-July), while in the moist transitional zone, almost half of the maize (49%), is produced in the minor season (October–February).

### 3.2 Knowledge and recognition of the FAW by maize-producing communities

**Fig. 2:**
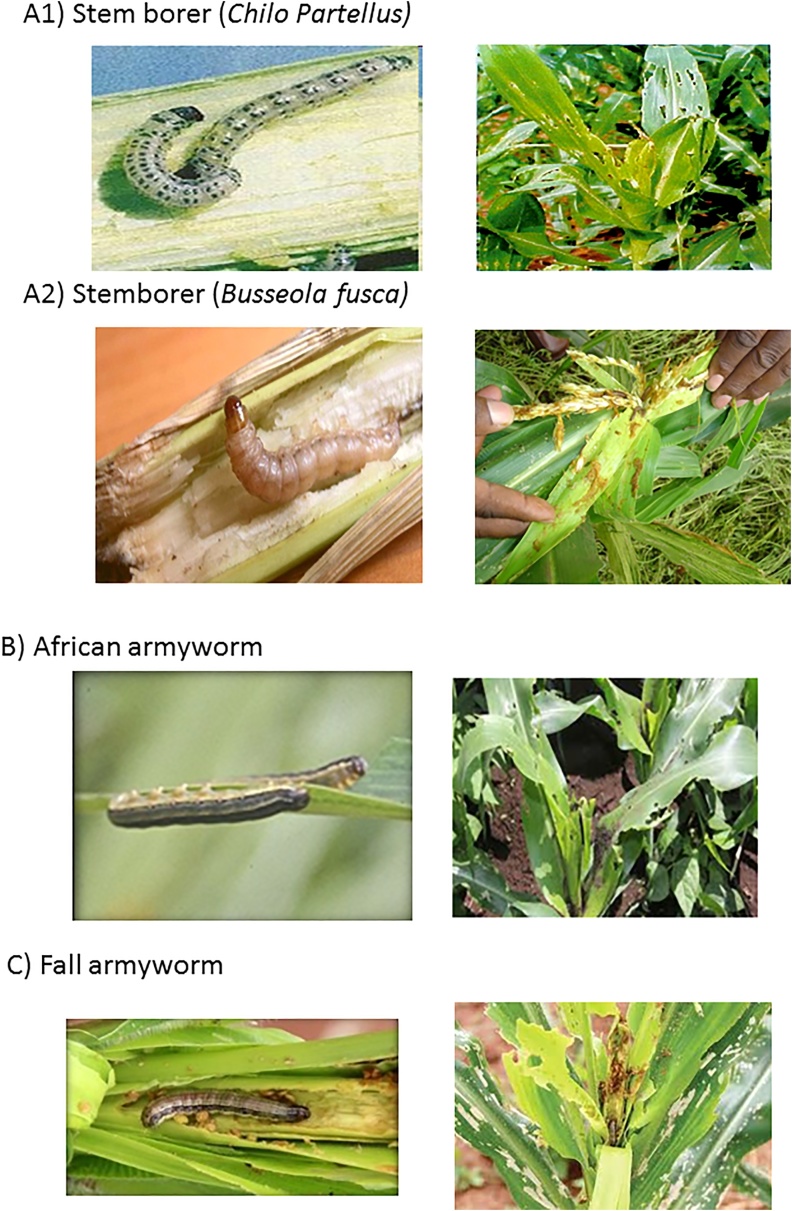
Pictures of lepidopterous insect pests shown to farmers: A) stemborers (either Chilo partellus, A1, or Busseola fusca, A2, depending on the zone); B) African armyworm; and C) fall armyworm.

Group discussions were held in 121 communities that were randomly selected and representative of the six major maize production zones in Kenya. In total, 1439 people participated with an average of 12 people per group, and slightly more than half of the participants (51%) were women. At the beginning of the discussions on the FAW, participants were shown six pictures of three different insects: stemborers, African armyworm (AAW) and fall armyworm (FAW), one picture with the larva and one with the damage for each insect (Fig. 2), and asked if they could recognize the FAW among them. Two stemborer species are common in Kenya: *Chilo partellus* (Swinhoe) in the lower areas and *Busseola fusca* (Fuller) in the highlands, but farmers do not generally distinguish between the two. Therefore, depending on the area, a picture of only one of the two stemborer species was shown (either Panel A1 or Panel A2).

Most participants (82%) could correctly identify the FAW from the images (Table 2). The percentage was slightly higher among women (84%) than among men (79%), although this was not consistent across agroecological zones. In Kenya, women are more involved in farm management than men (Kassie et al., 2014), which probably explains the reason why women have more knowledge of FAW than their male counterparts. Further, there were some differences among the zones, with the highest levels of correct responses found in the dry mid-altitude zones (94%) and the lowest in the highlands and coastal lowlands (both 76%).

**Table 2:** Percentage of participants who correctly identified FAW from pictures, by gender.

Gender	Agro ecological zones						Total	N
	Coastal Lowland	Dry mid altitude	Dry Transitional	Moist Transitional	High Tropics	Moist mid altitudes		
Women	65	85	88	89	83	88	84	742
Men	82	100	83	77	68	71	79	697
Total	76	94	85	84	76	80	82	1439

### 3.3 Arrival and spread of the FAW in Kenya’s maize production zones

**Fig. 3:**
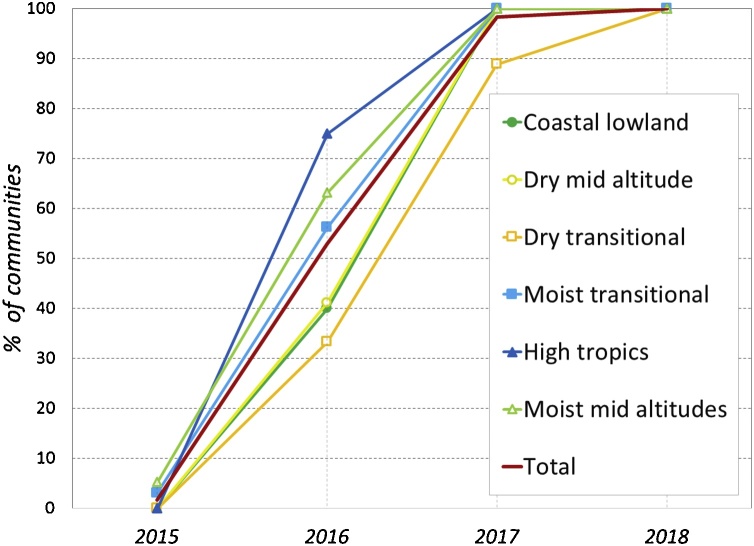
The spread of FAW, in cumulative % of communities that observed the pest over time.

After the identification exercise, participants were asked if they had observed the FAW in their community and, if so, in which year they had first observed it (Fig. 3). At the time of the survey (June-July 2018), the FAW had been observed in all the communities (either by the women or the men, or both). The first observations were made in 2015, although only by two communities (2%), while by the next year, 2016, FAW had been observed by half of the communities (51%). Most of the other half observed FAW first in 2017, with the remainder (2%) observing it first in 2018.

There was some difference in the arrival and spread between the different zones. FAW was first observed in 2015 in western Kenya (Fig. 3), followed by its sighting in high rainfall areas in the west and central Kenya, where more than half of the communities had observed it in 2016. In that year, first observations of FAW were more common in the highlands (75% of communities), followed by the moist mid-altitude (63%) and moist transitional zones (56%). In the dry areas and at the coast, most communities did not observe FAW until 2017.

These results show a very rapid spread of an insect pest, largely over a period of only two years, overall maize-growing areas in the country. Further, the year of the arrival of the FAW was positively correlated with longitude (in decimal degrees) (0.203, p = 0.025), and negatively with latitude (−0.160, p = 0.08), indicating a weak NW to SE movement over the three years.

### 3.4 Farmers affected and losses caused by FAW

**Fig. 4:**
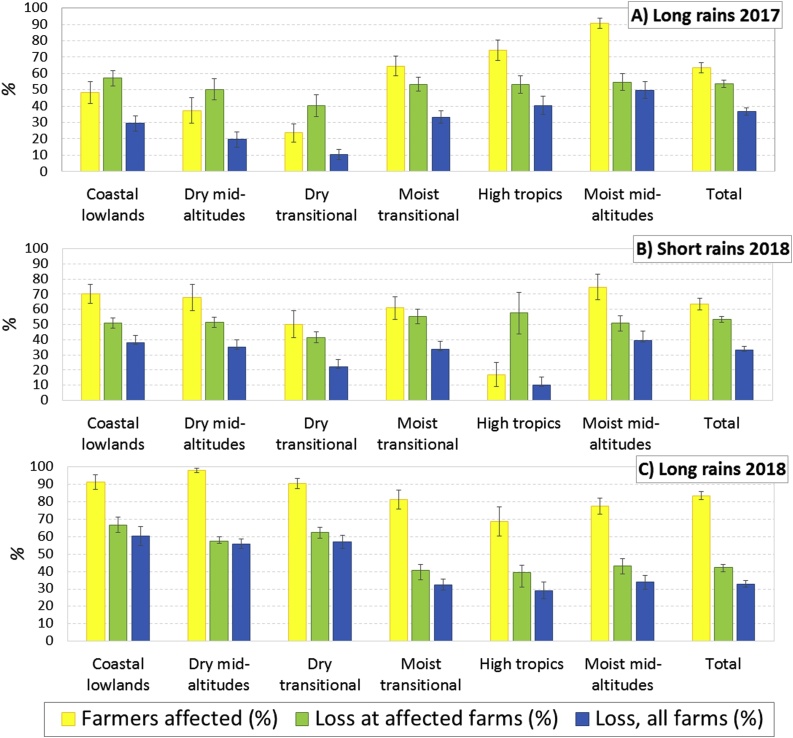
Percentage of farmers affected by FAW, maize crop loss among affected farmers (%) and maize crop loss among all farmers over the last three seasons, by AEZ (total is weighted by maize production in AEZ).

Next, participants were asked to estimate how many farmers in their community were affected, and what percentage of maize was lost on those farms, for the current season (long rains 2018) and for the last two seasons (short rains 2017 and long rains 2017). The results per maize production zone are presented in Fig. 4 (the numbers are provided in Appendix 3). To calculate the totals overall maize zones, we weighted the means of the percentage of farmers affected by the FAW population in that zone (Appendix 2), and we weighted the means of the losses by maize production in that zone, to calculate the overall mean for losses.

The results show that the proportion of farmers affected by FAW substantially increased from the long rains of 2017 (63%) to those of 2018 (83%), for all zones except for the high tropics and moist mid-altitudes. In the short rains of 2018, the proportion of farmers affected remained the same (63%), but the average masked large regional differences. In the low-potential maize zones (coast and drylands), the number of farmers affected increased substantially, while those in the highlands reduced because farmers there grow little maize in the short rainy season. In the last season, the long rains of 2018, 83% of farmers, and at least two-thirds of farmers in each zone, were affected.

The yield loss percentage among affected farmers decreased slightly over the three seasons, from 54% (LR 2017) over 53% (SR 2017) to 42% (LR 2018). The last number was an estimate, as the actual harvest of the LR 2018 had not yet taken place at the time of the survey. Total yield loss for each community was calculated by multiplying the proportion of farmers affected by their loss, and these were averaged for each zone. Total losses for each zone was calculated by multiplying the relative loss with the average production for that zone, per season, before the arrival of the FAW, and summed up for the national total (details in Table 3). The results showed a total loss of 37% in the first season (LR 2017), followed by 33% in the next two seasons (SR 2017 and LR 2019). The total yield loss more than doubled over the study period at the coast and in the dryland and highland areas, but stayed steady in the moist transitional and even reduced in the moist mid-altitude zones.

**Table 3:** Maize production and losses due to FAW by maize production zone.

Maize production zone	Average maize production		Long-Rains 2017		Short Rains 2017		Long Rains 2018	
	Long rains	Short rains	Average loss	Estimated loss	Average loss	Estimated loss	Estimated loss	Average loss
	(1000 tonnes)	(1000 tonnes)	(%)	(1000 tonnes)	(%)	(1000 tonnes)	(%)	(1000 tonnes)
Lowland Tropics	26	16	26	7	36	6	59	15
Dry Mid-altitude	17	24	14	2	32	8	56	10
Dry-Transitional	111	106	6	7	21	22	56	62
Moist-transitional	1081	383	29	317	31	120	32	345
Highlands	898	9	38	340	10	1	28	250
Moist Mid-altitude	279	180	50	139	39	70	34	94
Other	333	95	34	112	32	30	32	107
Grand total	2745	814	34	924	32	257	32	883

**Fig. 5:**
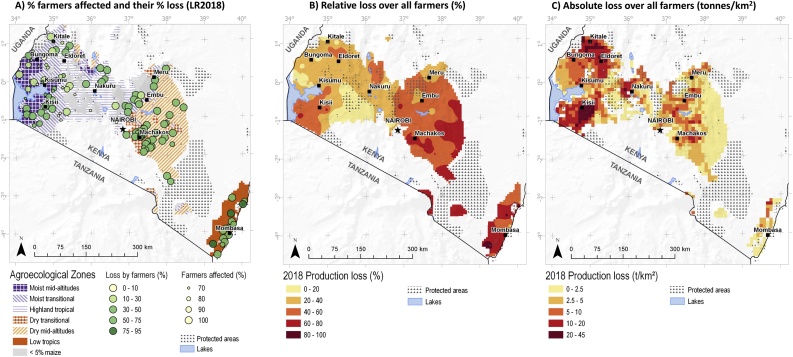
Geographic distribution of maize losses due to fall army worm: A) proportion of farmers affected and the % of maize lost by affected farmers during the long rains of 2018; B) distribution of relative losses among all farmers in % in 2018 (both seasons); C) distribution of absolute losses in 2018 in tonnes/km2 (both seasons).

As all communities were georeferenced, we can analyze the geospatial distribution of the impact of FAW (Fig. 5, Panel A: each circle represents a community surveyed, the size of the circle represents the % of farmers affected, the color their loss). By the long rains of 2018, almost all farmers were affected at the coast and in the drylands, where losses among affected farmers are also very high. In the highlands in Central Kenya, and to a lesser extent in the north-eastern major maize production areas, the proportion of farmers affected, as well as the losses caused among those farmers, are distinctly lower than in the South-East. The southwest (around Kisii), on the other hand, in particular, the southern moist mid-altitudes and the south-western moist transitional zones, on the other hand, were seriously affected. Similarly, the maize areas in the northwest (around Eldoret) were heavily affected.

Extrapolating the total maize losses (in percentages) in individual, georeferenced communities over the different agro-ecological zones over both seasons provided a detailed map with the distribution of those losses (Fig. 5, Panel B: distribution of relative losses, in %, among all farmers in 2018, over both seasons)). The map for 2018 allows us to identify several areas with proportionally high losses, in particular, the coastal lowlands, the southern drylands, the eastern moist transitional-, and the southern moist transitional- and moist mid-altitude zones. Proportionate losses are relatively low in the highlands (except for just north of Eldoret), and also in the northern moist transitional- and mid-altitude zones.

Multiplying proportionate losses with the actual maize production (derived from SPAM 2010) produced a map with absolute losses for the long rains of 2018, expressed in tonnes per km^2^ (Fig. 5, Panel C: distribution of absolute losses, in tonnes/km^2^ in 2018, over both seasons). Moving from relative to absolute losses, the picture does change substantially. While losses at the coast and in the drylands are relatively high, those zones do not produce much maize, and therefore the quantity of maize lost there is relatively small. In absolute terms, maize losses are high in four areas: in the east in the dry transitional and moist mid-altitude areas (from Machakos to Embu); in the west in the moist transitional zone in the south (Kisii), and in the north (Kisumu-Bungoma); and in the highlands from Kitale to Eldoret.

The communities were randomly selected to be representative of the AEZs. However, communities in less productive zones were oversampled, so the mean maize yield loss percentage of all communities is not representative of the country as a whole. Therefore, for each zone, the average relative loss is multiplied by the estimated production of that zone before the arrival of the FAW, to estimate the actual yield loss for each zone (Table 3). The absolute losses can then be aggregated over the zones to estimate the absolute nationwide loss, and divided by the total production before the arrival of the FAW to estimate the relative loss. Alternatively, total losses per AEZ could be calculated by adding up the production losses for each of the grids, but because of accuracy issues with the SPAM 2010 data, this procedure was not pursued.

The results show that, as most of the maize is produced in the main season, most of the losses also occur in that season. The estimated losses of maize production due to FAW come to 924,000 tonnes (34%) in the long rains of 2017, and 883,000 tonnes (32%) in the same season of 2018. As less maize is produced in the short rains, the absolute losses are smaller in that season. In the short rains of 2017, they are estimated at 257,000 tonnes. The relative losses in the short season, however, are 32%, similar to those in the long rains.

Further, the highest relative losses are found in the low-potential areas of the east, in particular for the long rains of 2018, when the losses were estimated at more than half. However, since most of the maize is produced in the high-potential zones of the west, most of the actual losses occur in these zones. In the long rains, the moist-transitional areas and highlands account for most of the losses: 71% in 2017 and 67% in 2018. In the short rains, on the other hand, losses in the highlands are negligible, but the moist-transitional zone still accounts for almost half (47%) of the losses.

## 4 Discussion

The results of our group discussions in the 121 communities, which are representative of the different agro-ecological zones in Kenya, show clearly that the FAW has become a major pest in a very short time. It arrived suddenly in 2015 or 2016, spread very fast over two years, and by 2018 affected most maize farmers in the country (86%). Only two communities observed FAW in 2015, and these were not confirmed by other sources, so these reports might have been subject to observational or recall error. An alternative explanation is that while the pest was first formally reported in Africa in 2016 (Goergen et al., 2016), it could have arrived earlier, and observed by some communities in 2015.

Extrapolating the estimates from the farmers over the production estimates of the different zones before the arrival of the FAW, we estimate the loss of maize due to the FAW at about one-third of the potential production. More specifically, our estimates are 34% in the long rains of 2017, and 32% in both the short rains of 2017 and the long rains of 2018. Substantial differences between the maize production zones were observed. In the long rains of 2017, the low- and medium-potential areas were most affected, with total losses of more than 50%, while losses in the high-potential areas (highlands and moist transitional) were much lower (about 30%), leading to an overall estimated loss of 34%. In the long rains of 2018, losses in the low- and medium-potential areas had reduced to about 20%, but the high-potential areas were now more affected, leading to an estimate of losses of 32%. In total, based on the farmer estimates and our calculations, we estimate that Kenya is losing about a third of its annual maize production, more than 1 million tonnes of maize.

In response to the severity of FAW attacks and the resulting losses, a range of control methods have been tested and disseminated. In Latin America, on large-scale farms, FAW is largely controlled by genetically modified organisms (GMOs) and insecticides (Burtet et al., 2017; Day et al., 2017). In Africa, however, most countries do not allow GMOs, and the use of pesticides is problematic education and knowledge about their risks and proper use (Baudron et al., 2019). In Africa, different studies have estimated the effectiveness of available control measures through farmer perceptions or biophysical observations. In Kenya, most farmers (60%) perceived pesticides as not effective in controlling FAW, compared with only a third (32%) in Ethiopia (Kumela et al., 2018). The effectiveness of ‘climate-adapted push-pull’ (CAPP) as a tool for managing FAW was assessed on 250 farms in drier areas of western Kenya, eastern Uganda and northern Tanzania for the March 2017 growing season (Midega et al., 2018); compared to maize monocropping, plant damage from fall armyworms when using CAPP was reduced by 86.7%, while maize grain yields were 2.7% higher. Farmers also rated the CAPP technology significantly superior in reducing FAW infestation and damage. A survey carried out on 35 farms in Uganda, using observations on the intensity of infestation (on a 1–5 scale) on randomly selected plots, showed that, in addition to push-pull, intercropping of maize with edible legumes could also be an alternative FAW management option (Hailu et al., 2018). A recent paper reviewing the literature on agroecological approaches from Central and South America concluded that several interventions were ready to be piloted at scale, including minimum tillage, biomass mulching, intercropping, and diversification of the farm environment through crop rotation and other measures (Harrison et al., 2019). In Zimbabwe, FAW infestation of maize was found to be significantly reduced by weed control (Baudron et al., 2019). Economic analysis is now needed to compare the loss abatement brought by the control methods to its costs, including not only input costs, but also labor costs and, especially for the chemical control methods, the costs to the environment and the health of the farm workers.

We found that participants in the group discussions were quite knowledgeable about FAW, especially the women. Women might be more knowledgeable than men because they are more involved in farmer management, especially in the vegetative stages when FAW damage is more visible. Women, for example, contribute 63% of labor for weeding and 52% for harvesting (Kassie et al., 2014). Interestingly, the two zones where men are more knowledgeable are also the warmest zones, were crops are more prone to insect attacks, which can make farmers more attentive to pests. The lowest, and warmest, three zones also recorded the highest proportion of farmers affected by FAW and the highest losses during the last season under study (Fig. 5). Temperature is known to be the dominant abiotic factor directly affecting herbivorous insects: it directly affects development, survival, range and abundance (Bale et al., 2002).

While the precision of the estimates can be calculated through standard errors (Fig. 5), it is much harder to assess their accuracy, as no alternative measurements are available. In particular, we did not conduct parallel direct observations of yield losses due to FAW, as that was not within the objectives and the budget for the study, so farmers’ estimates could not be verified. However, several factors support the estimates presented here. First, participants established good recognition of the FAW. Among pictures of the larvae of FAW, African armyworm and stemborers, most participants (82%) could correctly identify FAW. Second, in previous research on farmer estimates of losses from insect pests, the farmer estimates of losses of maize to stemborers were very close to direct observations: 12.9% as estimated by farmers (De Groote, 2002) versus 13.5 % in direct observations (De Groote et al., 2004a). In comparison, the calculation for losses due to stemborers from this community survey, calculated in a similar fashion as for FAW, resulted in estimates of 13%, 11% and 7% in the three consecutive seasons. This would indicate that farmers are not likely to overestimate losses from insect pests, or at least not from stemborers, a pest they are very familiar with and which leaves distinct damage marks on the maize leaves. Third, our results, which show about a third of the harvest lost, agree with other estimates, from two household socio-economic surveys (Abrahams et al., 2017; Kumela et al., 2018). The first study, conducted in July 2017 in Ghana and Zambia, using household socio-economic surveys, estimated the perceived yield losses due to FAW by maize farmers at 40% (range 25–50%*)* and 45% *(*22–67%), respectively. Extrapolation of the results indicated that FAW in Africa had the potential to cause maize yield losses from 8.3 to 20.6 million tonnes per annum (21–53% of production), valued at US$ 2.4–6.2 billion, if left uncontrolled (Abrahams et al., 2017; Day et al., 2017). In a second study, farmers’ perceptions were elicited in 2017 from areas in East Africa where the FAW had been reported, based on surveys of randomly selected farmers in purposively selected districts (150 farmers from three districts in Ethiopia, and 193 farmers from five sub-counties in Kenya) (Kumela et al., 2018). On average, farmers estimated infestation rates of 32% in Ethiopia and 47% in Kenya, with estimated maize yield reductions of 934 kg/ha in Ethiopia and 1381 kg/ha in Kenya. These numbers are based on estimates by farmers or experts, not on direct measurements.

Admittedly, our results diverge from the only actual measurements of losses due to FAW in Africa that we are aware of, namely, a study in Zimbabwe where yield loss was estimated at 11.6% (Baudron et al., 2019). That estimate was, however, based on data from only one season in two districts of one region. In our study, we found a similar low level of losses only in the highlands in the short rains of 2017. The authors of the study argue that loss assessment based on farmer perception could overestimate the real losses. At this stage, this argument remains valid, especially with the sudden arrival of a dramatic invasive pest like FAW. The discussion can only be settled through a rigorous comparison of field trials with farmer estimates, preferably both with individual farmers and through community surveys.

Despite the discussion about the accuracy, our experience shows that community surveys are a convenient tool for conducting a quick, and cost-efficient survey for an invasive pest. Figures on the cost of surveys are rarely published, but they are essential for comparing costs to accuracy (De Groote, 1996). This survey took six weeks to reach 121 communities, working in two independent teams, and cost $50,000, including the cost of data collection and preliminary analysis, but not the scientists’ time for preparation, final analysis, and write-up.

We conclude that the FAW is an important pest that arrived suddenly and spread very quickly, destroying about a third of the harvest and that farmers estimate the losses it causes at about one-third of their maize crop. Farmers have difficulties coping with this pest, and appropriate control measures are urgently needed.

## Funding

This research was supported by the Bill and Melinda Gates Foundation (BMGF), Howard G. Buffett Foundation and the United States Agency for International Development (USAID), through the Stress Tolerant Maize for Africa (BMGF Opportunity/Contract ID OPP1134248 and USAID Grant ID MTO 069033), the CGIAR Research Program MAIZE, and the project “Integrated pest management strategy to counter the threat of invasive fall armyworm to food security in Eastern Africa” (FAW-IPM, Grant # DCI- FOOD/2018/402-634), funded by EU. We also gratefully acknowledge core financial assistance to the International Centre of Insect Physiology and Ecology (*icipe*) provided by the United Kingdom’s Department for International Development (DFID).

## Declaration of Competing Interest

The authors declare that they have no known competing financial interests or personal relationships that could have appeared to influence the work reported in this paper.
