# Supplementary material for: Spread and impact of fall armyworm (Spodoptera frugiperda J.E. Smith) in maize production areas of Kenya
Source: Agric Ecosyst Environ. 2020 Apr 15;292:106804. doi: 10.1016/j.agee.2019.106804 (PMC7015277; doi:10.1016/j.agee.2019.106804)
Supplement: Supplementary file 2 [file mmc2.docx]

Appendix 2. Maize agroecological zones in Kenya, with estimated maize area and production in 1992, 2005 and 2010

| Agroecological zone | Elevation |  | Hassan (household survey 1992) | | | | SPAM 2005 | | | |  | | SPAM 2010 | | | |  | Population |  | Weights | | | |
| --- | --- | --- | --- | --- | --- | --- | --- | --- | --- | --- | --- | --- | --- | --- | --- | --- | --- | --- | --- | --- | --- | --- | --- |
|  |  |  | Area (1000 ha) | Prod. (1000 tonnes) | Yield (t/ha) |  | | Area (1000 ha) | Prod.  (1000 tonnes) | Yield (t/ha) | |  | | Area (1000 ha) | Production (1000 tonnes) | Yield (t/ha) |  | (1000) |  | Area | Prod. | Pop. |  |
| Lowland Tropics | 0-700 |  | 41 | 53 | 1.29 |  | | 47 | 37 | 0.77 | |  | | 56 | 30 | 0.54 |  | 2,857 |  | 0.03 | 0.01 | 0.06 |  |
| Dry Mid-altitude | 700-1400 |  | 166 | 162 | 0.98 |  | | 320 | 170 | 0.53 | |  | | 386 | 196 | 0.51 |  | 3,825 |  | 0.19 | 0.08 | 0.08 |  |
| Dry-Transitional | 1100-1700 |  | 66 | 76 | 1.15 |  | | 79 | 35 | 0.44 | |  | | 566 | 486 | 0.86 |  | 5,403 |  | 0.28 | 0.20 | 0.12 |  |
| Moist-transitional | 1200-2000 |  | 466 | 1234 | 2.65 |  | | 499 | 1,219 | 2.44 | |  | | 372 | 524 | 1.41 |  | 7,931 |  | 0.19 | 0.22 | 0.17 |  |
| Highlands | 1600-2900 |  | 316 | 909 | 2.88 |  | | 347 | 810 | 2.33 | |  | | 239 | 586 | 2.45 |  | 1,801 |  | 0.12 | 0.25 | 0.04 |  |
| Moist Mid-altitude | 1110-1500 |  | 173 | 231 | 1.34 |  | | 173 | 357 | 2.06 | |  | | 99 | 109 | 1.10 |  | 12,137 |  | 0.05 | 0.05 | 0.26 |  |
| < 5% |  |  |  |  |  |  | | 67 | 130 | 1.94 | |  | | 88 | 119 | 1.57 |  | 1,858 |  | 0.04 | 0.05 | 0.04 |  |
| Other |  |  |  |  |  |  | | 141 | 169 | 1.20 | |  | | 202 | 326 | 0.86 |  | 10,076 |  | 0.10 | 0.14 | 0.22 |  |
| Total |  |  | 1244 | 2671 | 2.1471 |  | | 1,674 | 2,927 | 1.75 | |  | | 2,007 | 2,376 | 1.18 |  | 45,890 |  | 1.00 | 1.00 | 1.00 |  |
