# Supplementary material for: Spread and impact of fall armyworm (Spodoptera frugiperda J.E. Smith) in maize production areas of Kenya
Source: Agric Ecosyst Environ. 2020 Apr 15;292:106804. doi: 10.1016/j.agee.2019.106804 (PMC7015277; doi:10.1016/j.agee.2019.106804)
Supplement: Supplementary file 3 [file mmc3.docx]

Appendix 3. Farmers affected, loss among affected farmers, and losses among all farms.

| Statistic | Agroecological zone | Long rains 2017 | | |  | Short rains 2018 | | |  |  | Long rains 2018 | | |
| --- | --- | --- | --- | --- | --- | --- | --- | --- | --- | --- | --- | --- | --- |
|  |  | Farmers affected (%) | Loss at affected farms (%) | Loss, all farms (%) |  | Farmers affected  (%) | Loss at affected farms (%) | Loss, all farms (%) |  |  | Farmers affected (%) | Loss at affected farms (%) | Loss, all farms (%) |
| Mean | Coastal lowlands | 48.5 | 57.1 | 29.4 |  | 70.2 | 51.1 | 37.9 |  |  | 91.3 | 66.5 | 60.4 |
|  | Dry mid-altitudes | 37.4 | 50.2 | 19.6 |  | 67.6 | 51.6 | 34.9 |  |  | 97.9 | 57.2 | 55.9 |
|  | Dry transitional | 23.6 | 40.2 | 10.5 |  | 50.2 | 41.5 | 22.1 |  |  | 90.4 | 62.3 | 56.9 |
|  | Moist transitional | 64.6 | 53.4 | 33.2 |  | 60.9 | 55.2 | 33.6 |  |  | 81.2 | 40.7 | 32.4 |
|  | High tropics | 74.3 | 53.2 | 40.4 |  | 17.0 | 57.5 | 10.1 |  |  | 68.8 | 39.5 | 29.0 |
|  | Moist mid-altitudes | 90.5 | 54.7 | 49.8 |  | 74.7 | 50.8 | 39.6 |  |  | 77.4 | 43.0 | 33.8 |
|  | Total (weighted) | 63.4 | 53.6 | 36.6 |  | 63.3 | 53.4 | 33.2 |  |  | 83.4 | 42.4 | 32.9 |
| SE | Coastal lowlands | 6.7 | 4.7 | 4.9 |  | 6.0 | 3.3 | 4.9 |  |  | 4.3 | 4.9 | 5.3 |
|  | Dry mid-altitudes | 7.9 | 6.5 | 4.7 |  | 8.7 | 3.3 | 5.0 |  |  | 1.2 | 2.6 | 2.6 |
|  | Dry transitional | 5.4 | 6.7 | 3.2 |  | 8.9 | 3.4 | 4.8 |  |  | 3.1 | 3.1 | 3.8 |
|  | Moist transitional | 6.1 | 4.4 | 3.8 |  | 7.6 | 4.8 | 5.2 |  |  | 5.4 | 3.2 | 3.2 |
|  | High tropics | 6.1 | 5.2 | 5.6 |  | 8.1 | 13.6 | 5.5 |  |  | 8.4 | 4.2 | 4.8 |
|  | Moist mid-altitudes | 3.1 | 5.1 | 5.1 |  | 8.5 | 5.0 | 6.1 |  |  | 4.6 | 4.2 | 3.9 |
|  | Total | 3.2 | 2.2 | 2.2 |  | 3.7 | 1.9 | 2.4 |  |  | 2.4 | 1.8 | 2.0 |
| SD | Coastal lowlands | 25.9 | 17.4 | 18.8 |  | 23.4 | 12.7 | 18.9 |  |  | 16.7 | 18.8 | 20.5 |
|  | Dry mid-altitudes | 32.4 | 22.6 | 19.5 |  | 35.7 | 12.9 | 20.6 |  |  | 5.0 | 10.7 | 10.6 |
|  | Dry transitional | 22.9 | 23.1 | 13.8 |  | 37.8 | 13.3 | 20.2 |  |  | 13.2 | 13.0 | 16.3 |
|  | Moist transitional | 34.3 | 22.6 | 21.4 |  | 42.7 | 22.9 | 29.3 |  |  | 30.4 | 17.3 | 18.3 |
|  | High tropics | 27.4 | 22.8 | 25.1 |  | 36.1 | 27.2 | 24.5 |  |  | 37.7 | 17.4 | 21.4 |
|  | Moist mid-altitudes | 13.4 | 22.4 | 22.3 |  | 37.1 | 20.1 | 26.5 |  |  | 20.0 | 18.3 | 17.2 |
|  | Total | 34.9 | 22.0 | 23.8 |  | 41.1 | 18.2 | 26.1 |  |  | 25.9 | 19.2 | 21.7 |
| N | Coastal lowlands | 15 | 14 | 15 |  | 15 | 15 | 15 |  |  | 15 | 15 | 15 |
|  | Dry mid-altitudes | 17 | 12 | 17 |  | 17 | 15 | 17 |  |  | 17 | 17 | 17 |
|  | Dry transitional | 18 | 12 | 18 |  | 18 | 15 | 18 |  |  | 18 | 18 | 18 |
|  | Moist transitional | 32 | 27 | 32 |  | 32 | 23 | 32 |  |  | 32 | 29 | 32 |
|  | High tropics | 20 | 19 | 20 |  | 20 | 4 | 20 |  |  | 20 | 17 | 20 |
|  | Moist mid-altitudes | 19 | 19 | 19 |  | 19 | 16 | 19 |  |  | 19 | 19 | 19 |
|  | Total | 121 | 103 | 121 |  | 121 | 88 | 121 |  |  | 121 | 115 | 121 |

SE = standard error, SD = standard deviation, N = number of communities
